# Supplementary figures and images for: Application of type II diabetes incidence and mortality rates for insurance
Source: PLoS One. 2024 Sep 25;19(9):e0307508. doi: 10.1371/journal.pone.0307508 (PMC11423961; doi:10.1371/journal.pone.0307508)

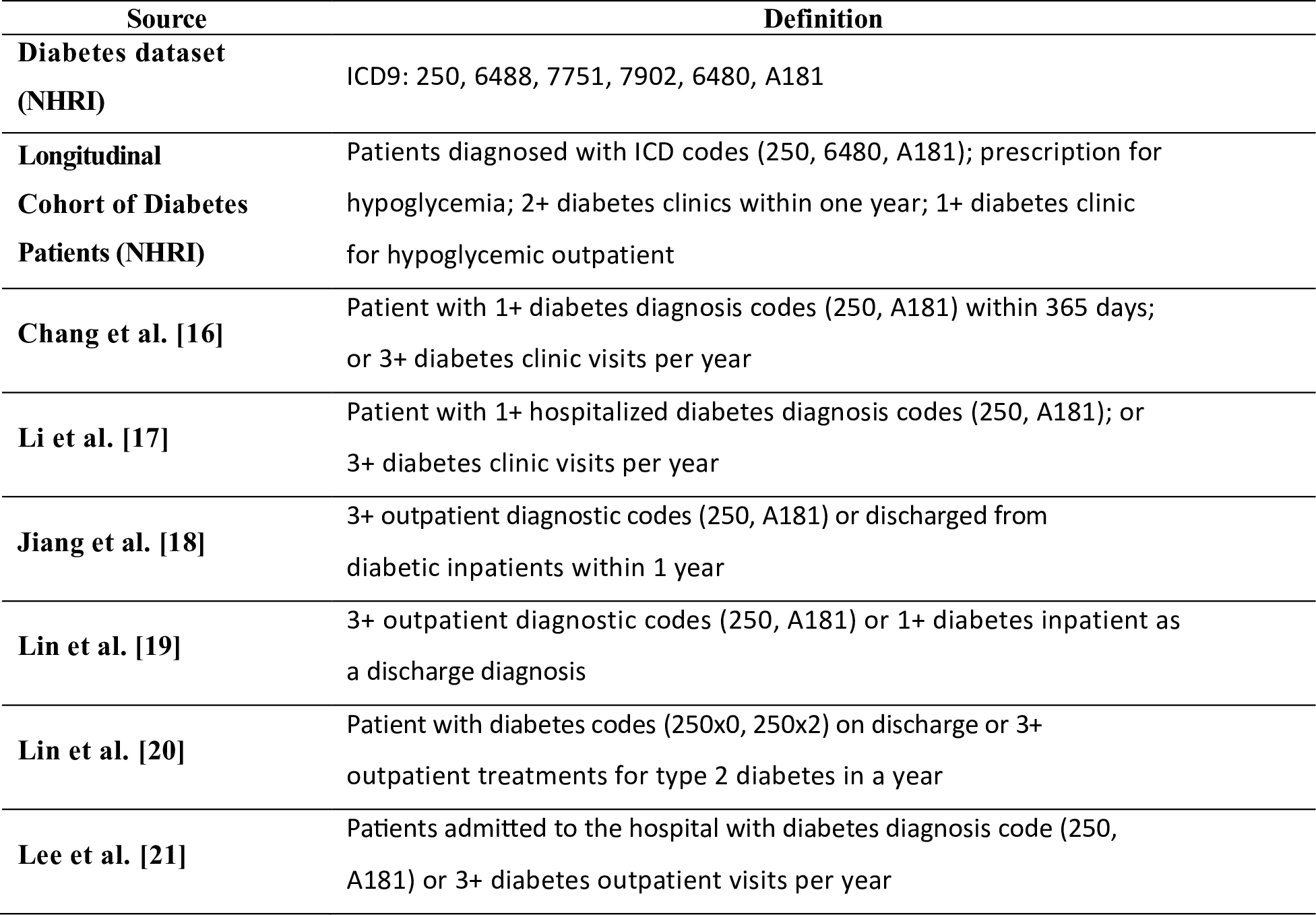

Supplement: S1 Table — (TIFF) [file pone.0307508.s001.tiff]
