# Supplementary material for: Application of type II diabetes incidence and mortality rates for insurance
Source: PLoS One. 2024 Sep 25;19(9):e0307508. doi: 10.1371/journal.pone.0307508 (PMC11423961; doi:10.1371/journal.pone.0307508)
Supplement: Attachment — Submitted filename: Communications_T2DM for Insurance (20240406).docx [file pone.0307508.s002.docx]

Dear Editor,

This is in reply to your letter of Feb. 10, 2024 regarding our manuscript, Application of Type II Diabetes Incidence and Mortality Rates for Insurance. We appreciate the helpful and insightful comments from the anonymous referees. We have revised the manuscript substantially in light of these comments (marked in red), and hereby enclose a copy of the revised manuscript.

**Reviewer #1**

**Major Comments**

1. The structure of the paper should follow the conventional order of Materials and Methods, Results, Discussion, and Conclusions. This would make the paper more coherent and easier to follow. Currently, the paper seems to mix the discussion with other sections, which reduces the impact of the main findings.

**Response:**

We have revised the order of our manuscript according to the reviewer’s suggestion, and the manuscript follows the order of Materials and Methods, Results, Discussion, and Conclusions.

2. The introduction should be more concise and focused on the rationale and objectives of this specific study. There is too much background information on general trends in population aging, chronic disease, and diabetes prevalence that may not be directly relevant to the research question. The introduction should clearly state the research gap, the hypothesis, and the contribution of the paper.

**Response:**

We appreciate the reviewer’s comment and thus have revised the introduction, making it more concise and focused. In addition to removing unnecessary background information, we state the research gap, the hypothesis, and the contribution of the paper in the penultimate paragraph of the introduction.

3. Generally, the context should also be more concise and contain only relevant information to this study. For example, lines 263-268 use too many sentences/words to introduce RP and its benefits, which are irrelevant to this study.

**Response:**

We have revised the manuscript according to the reviewer’s suggestion, making the paper more concise and containing only relevant information.

4. The discussion should elaborate more on the implications of the findings and limitations. For example, how could the data inform specific insurance product designs or public health policies? What are the potential benefits and challenges of using such data for insurance purposes? What are the limitations of claims data for identifying diabetes cases and measuring outcomes? How do the results compare with other studies in the literature?

**Response:**

We appreciate the reviewer’s comments. This study has three main limitations, including the data period, the definition of diabetes, and the death criteria. We use only the data sets up until 2013, since there is a time gap in the release of diabetes data and Taiwan implemented the Personal Data Protection Act in 2012, increasing the difficulty of human-related research. Also, as mentioned in the materials and methods section, the definition of diabetes is controversial and thus we use the outpatient records to judge whether a person has diabetes. Our study results may not be applied to other studies if different diabetes criteria are sued. Similarly, the mortality rates of diabetes patients are determined based on medical records, which may be different from the official statistics or other studies. For example, previous studies showed that older diabetes patients have higher mortality rates. We have added the preceding description in the discussion section.

As for the implication of this study, Diabetes is generally thought to be associated with a higher mortality rate in Taiwan and diabetes patients are not easy to purchase life insurance products. Our analysis results show that if patients with diabetes continue to receive treatment, their mortality rates are not much different from those of the general population. In other words, we applied the idea of big data to verify that people with diabetes do not necessarily live shorter. It provides new thinking for insurance companies in product design, as well as giving policyholders more opportunities to purchase insurance products.

**Minor Comments**

1. Use abbreviations consistently, e.g. T2DM vs. type 2 diabetes. Define the abbreviations at the first occurrence and use them throughout the paper.

**Response:**

We have defined the abbreviation at the first occurrence and use them throughout the manuscript.

2. In line 175, this is the first time “SMR” shows up, please define it there.

**Response:**

We have added the definition of SMR when it was first mentioned.

3. In lines 181-183, does SMR being “larger” than 1 indicate higher mortality rates?

**Response:**

We apologize for the error and have corrected it.

4. In lines 288-289, please supplement the results using criteria 2 and 3 outpatient visits per year as a reference.

**Response:**

We appreciate the reviewer’s suggestion. In this article, we mainly focus on the rates estimated under the criteria of 4 outpatient visits per year, but we still calculate those under other criteria. We found that the diabetes prevalence rates under criteria 2 and 3 outpatient visits per year results are slightly larger than those under 4 outpatient visits per year. For example, Figure A-1 shows the diabetes prevalence rates under 2 and 4 outpatient visits per year.

Fig A-1. Diabetes Prevalence Rates of 2 and 4 Outpatient Visits per Year (2008-12)

5. In line 325, please define PSMR.

**Response:**

We appreciate the reviewer’s comment and we have added the definition of PSMR in lines 173-178.

6. In lines 334-335, please provide the result when you omit the data in 2005 and 2006 as supplementary data. What is the reason why omitting the data in these two years would result in satisfactory fitting results?

**Response:**

We appreciate the reviewer’s suggestion. We propose an observation period of two years for determining if a person had diabetes to avoid moral hazard. The analysis results (Table 2) support our idea and omitting the years 2005 and 2006 would result in more stable diabetes incidence rates. In addition, the following graph (Fig A.2) show the male age-specific incidence rates of diabetes, 2005~2012, and the results of 2005 and 2006 look different than those of other years. Due to space limitations, this figure is not included in the article.

Incidence Rates

| Age | 2005 | 2006 | 2007 | 2008 | 2009 | 2010 | 2011 | 2012 |
| --- | --- | --- | --- | --- | --- | --- | --- | --- |
| 45 | 0.00235787 | 0.00391798 | 0.00586211 | 0.00519185 | 0.00711054 | 0.00779989 | 0.0080656 | 0.00683761 |
| 46 | 0.00169249 | 0.00387856 | 0.00662691 | 0.00685109 | 0.00884049 | 0.00735198 | 0.00800869 | 0.00750648 |
| 47 | 0.00152102 | 0.00479808 | 0.00691057 | 0.00808843 | 0.00683222 | 0.00875016 | 0.009996 | 0.00996264 |
| 48 | 0.00235433 | 0.00466102 | 0.00673491 | 0.0117452 | 0.0101523 | 0.00902935 | 0.00862527 | 0.00841934 |
| 49 | 0.0024134 | 0.00680066 | 0.0081979 | 0.010079 | 0.0092997 | 0.0114143 | 0.0106483 | 0.00877785 |
| 50 | 0.0024945 | 0.00520607 | 0.00890253 | 0.00641212 | 0.00820584 | 0.0106337 | 0.0118829 | 0.0118408 |
| 51 | 0.00221134 | 0.00631389 | 0.00968878 | 0.0101404 | 0.0119858 | 0.0122507 | 0.00925518 | 0.012095 |
| 52 | 0.00329381 | 0.00698846 | 0.00981294 | 0.00899281 | 0.0106773 | 0.0119715 | 0.0138646 | 0.00937779 |
| 53 | 0.00338009 | 0.00657562 | 0.00939199 | 0.0103448 | 0.0137594 | 0.0152845 | 0.0111145 | 0.011281 |
| 54 | 0.00410256 | 0.00571528 | 0.0108378 | 0.0114343 | 0.0157668 | 0.0152157 | 0.0141219 | 0.014989 |
| 55 | 0.00419727 | 0.00773694 | 0.0116279 | 0.0108886 | 0.0131895 | 0.0147679 | 0.0153748 | 0.0130663 |
| 56 | 0.00433098 | 0.0114669 | 0.0118769 | 0.0123767 | 0.0163934 | 0.0136115 | 0.0134933 | 0.014232 |
| 57 | 0.00369731 | 0.00919161 | 0.0150709 | 0.0170893 | 0.0157466 | 0.0156624 | 0.0173004 | 0.0171856 |
| 58 | 0.00473934 | 0.00785805 | 0.00961076 | 0.0149321 | 0.0171181 | 0.0160153 | 0.0132125 | 0.0188644 |
| 59 | 0.00515274 | 0.00918836 | 0.0148864 | 0.0123609 | 0.0183721 | 0.0174893 | 0.014786 | 0.0175067 |
| 60 | 0.00675676 | 0.00906687 | 0.0141643 | 0.017149 | 0.0229046 | 0.0180636 | 0.0180879 | 0.016939 |
| 61 | 0.00418848 | 0.0148536 | 0.0119645 | 0.0158473 | 0.0167215 | 0.0177685 | 0.0206186 | 0.0154786 |
| 62 | 0.00501792 | 0.00612392 | 0.0166817 | 0.0173776 | 0.0182421 | 0.0189212 | 0.0179866 | 0.0193286 |
| 63 | 0.00405605 | 0.013289 | 0.0117734 | 0.016752 | 0.0204583 | 0.0197682 | 0.0180495 | 0.0171888 |
| 64 | 0.00219941 | 0.00874525 | 0.0117114 | 0.0227273 | 0.0211844 | 0.0185968 | 0.0246566 | 0.0186354 |
| 65 | 0.00293686 | 0.0124575 | 0.0148437 | 0.0186698 | 0.0179758 | 0.0154614 | 0.0184778 | 0.0199637 |
| 66 | 0.00451863 | 0.00988217 | 0.0121473 | 0.0165123 | 0.0201207 | 0.0193783 | 0.0191215 | 0.0221019 |
| 67 | 0.00451549 | 0.0115771 | 0.0110367 | 0.0164395 | 0.0195021 | 0.0188521 | 0.0247587 | 0.028312 |
| 68 | 0.00476364 | 0.00922708 | 0.0139636 | 0.0183225 | 0.0152893 | 0.0232759 | 0.0191221 | 0.0195908 |
| 69 | 0.0040413 | 0.0097071 | 0.0124706 | 0.0189435 | 0.0217206 | 0.0206807 | 0.0208426 | 0.0188594 |
| 70 | 0.0049066 | 0.00986645 | 0.0127364 | 0.0157761 | 0.0166351 | 0.0251323 | 0.0237668 | 0.0243902 |
| 71 | 0.00466489 | 0.0103383 | 0.0136054 | 0.0172626 | 0.0184662 | 0.0189493 | 0.0197338 | 0.02058 |
| 72 | 0.00459796 | 0.011843 | 0.012968 | 0.0155065 | 0.0180787 | 0.020348 | 0.0202283 | 0.0190567 |
| 73 | 0.00350005 | 0.00890042 | 0.0139946 | 0.0161406 | 0.018258 | 0.0214394 | 0.0201277 | 0.0207944 |
| 74 | 0.00333612 | 0.0106089 | 0.0140427 | 0.0182781 | 0.0177669 | 0.0208134 | 0.0198326 | 0.019452 |
| 75 | 0.00464653 | 0.00849584 | 0.0143203 | 0.0155881 | 0.0188666 | 0.0185702 | 0.0193048 | 0.0185185 |
| 76 | 0.00355096 | 0.00968242 | 0.0131206 | 0.0146747 | 0.0154996 | 0.0180579 | 0.0180736 | 0.0188531 |
| 77 | 0.00323487 | 0.00830522 | 0.0125617 | 0.0148526 | 0.0171742 | 0.0178705 | 0.0197093 | 0.0192319 |
| 78 | 0.00423206 | 0.00807667 | 0.0111306 | 0.015132 | 0.0157019 | 0.0163984 | 0.0199262 | 0.0173175 |
| 79 | 0.00364987 | 0.0079574 | 0.0102471 | 0.0146794 | 0.0166025 | 0.0151532 | 0.0163572 | 0.0183478 |
| 80 | 0.00421911 | 0.00749186 | 0.0115739 | 0.013824 | 0.0154646 | 0.0154801 | 0.0170021 | 0.0193091 |
| 81 | 0.00465484 | 0.00705239 | 0.0111716 | 0.0142847 | 0.0162372 | 0.016178 | 0.0174149 | 0.0178709 |
| 82 | 0.00221262 | 0.00689841 | 0.0106062 | 0.0116305 | 0.0139063 | 0.0140034 | 0.017416 | 0.0173092 |
| 83 | 0.00316635 | 0.00663243 | 0.011185 | 0.0123498 | 0.0128958 | 0.015187 | 0.0180096 | 0.0156312 |
| 84 | 0.00186667 | 0.00746869 | 0.00964479 | 0.0128855 | 0.0121575 | 0.014631 | 0.0172582 | 0.0133424 |
| 85 | 0.00205233 | 0.00582183 | 0.00862944 | 0.0107443 | 0.0119033 | 0.014756 | 0.0135311 | 0.0152065 |
| 86 | 0.00169312 | 0.00709957 | 0.00976709 | 0.0107873 | 0.0124077 | 0.0113834 | 0.0133303 | 0.0140406 |
| 87 | 0.00246171 | 0.0047712 | 0.00674894 | 0.0106096 | 0.00972763 | 0.0131012 | 0.0128379 | 0.0143097 |
| 88 | 0.00171585 | 0.0049903 | 0.00826647 | 0.0109914 | 0.00867337 | 0.0121065 | 0.00946074 | 0.0118414 |
| 89 | 0.00183486 | 0.0035014 | 0.00954806 | 0.00804885 | 0.00812795 | 0.0140005 | 0.0105809 | 0.0116152 |

Fig A-2. Estimated Age-specific Incidence Rates of Diabetes (Male, 2005~2012)

**Reviewer #2**

**Major Comments**

1. The use of “metabolic syndrome” in the abstract (lines 31-32) requires clarification. This term is typically defined as a group of conditions increasing the risk of heart disease, stroke, and diabetes, and should not be conflated with these diseases themselves. People who have heart disease, stroke, or diabetes do not necessarily have metabolic syndrome.

**Response:**

We appreciate the reviewer’s suggestion and have revised the abstract.

2. Please list the limitations of your study in the discussion section.

**Response:**

We appreciate the reviewer’s suggestion regarding the limitations of our study. This study has three main limitations, including the data period, the definition of diabetes, and the death criteria. We use only the data sets up until 2013, since there is a time gap in the release of diabetes data and Taiwan implemented the Personal Data Protection Act in 2012, increasing the difficulty of human-related research. Also, as mentioned in the materials and methods section, the definition of diabetes is controversial and thus we use the outpatient records to judge whether a person has diabetes. Our study results may not be applied to other studies if different diabetes criteria are sued. Similarly, the mortality rates of diabetes patients are determined based on medical records, which may be different from the official statistics. We have added the preceding description in the discussion section.

3. The data sets you used only cover up until 2013, and there have been significant advancements in diabetes treatment since that time.

**Response:**

We appreciate the reviewer’s comments regarding the data used. There is a time gap in the release of Taiwan’s diabetes data, including National Health Insurance data. For example, Tsai and Li (2023) explored whether daily diet is related to the risk of type 2 diabetes in Taiwan and they used 2013-2016 data. Annals of Diabetes Mellitus in Taiwan 2019 Update on Type 2 Diabetes, published by Diabetes Association of the Republic of China (Taiwan), were based on the data from 2014 and before. Also, Taiwan implemented the Personal Data Protection Act in 2012, and there have been more and more restrictions on data application and use, making human-related research more difficult. As for the issue of diabetes treatment, diabetes has been one of the top ten causes of death in Taiwan for more than 30 years, and about 6% of annual deaths are diabetes related in past 15 years. Diabetes continues to poses a serious threat to Taiwan’s people, and treatments still need to be developed.

- Tsai TJ and Li MC. Adherence to the Taiwan Daily Food Guide and the Risk of Type 2 Diabetes: A Populational Study in Taiwan. International Journal of Environmental Research and Public Health. 2023 Feb; 20(3): 2246.

4. The implications of your findings for insurance product design are interesting. Further discussion could explore how these findings might practically influence current insurance models and their implications for policyholders and insurance companies.

**Response:**

We appreciate the reviewer’s suggestion and we have included a discussion of possible impacts of our findings to Taiwan’s insurance industry. Diabetes is generally thought to be associated with a higher mortality rate in Taiwan and the persons with diabetes are not easy to purchase life insurance. Our analysis results show that if patients with diabetes continue to receive treatment, their mortality rates are not much different from those of the general population. This discovery can be regarded as an application of big data. In addition, it provides new thinking for insurance companies in product design and gives policyholders more opportunities to purchase insurance products. We have added the preceding description in the conclusion section.
