# Supplementary material for: Application of type II diabetes incidence and mortality rates for insurance
Source: PLoS One. 2024 Sep 25;19(9):e0307508. doi: 10.1371/journal.pone.0307508 (PMC11423961; doi:10.1371/journal.pone.0307508)
Supplement: Attachment — Submitted filename: Communications_T2DM__for_Insurance (20240607).docx [file pone.0307508.s003.docx]

Dear Editor,

This is in reply to your letter of May 9, 2024 regarding our manuscript, Application of Type II Diabetes Incidence and Mortality Rates for Insurance. We appreciate the helpful and insightful comments from the anonymous referees. We have revised the manuscript in light of these comments (marked in red), and hereby enclose a copy of the revised manuscript.

**Reviewer #1**

**Major Comments**

1. Consider hiring an official English editor to improve the language quality. While your manuscript is generally well-written, a professional review could further enhance its readability. For example,

- In the abstract, references are generally not necessary. For instance, please review lines 35 and 39.

- In line 51, the hyphen in “Be-coming” is redundant. You can simply use “Becoming.”

- Regarding line 57, you can choose either “people ages 65 and over” or simply “elderly.” The parentheses provide no additional explanation.

- Throughout the paper, there is an overuse of the future tense (“will”). Please ensure consistency with the actual actions performed during your study to improve overall composition.

**Response:**

The previous version of our manuscript was proofread by a professional English editor, however, we hired another English editor to review the revised manuscript. Additionally, we have removed the references in the abstract and revised the incorrect words, such as “elderly” and “will.”

2. I commend your efforts to make the manuscript well-structured and concise. However, there are instances where statements seem misplaced:

- In the “Materials and Methods” section (lines 105-108), you mention that the National Health Insurance Research Database (NHIRD) covers about 99.6% of Taiwan’s citizens. While this information underscores the strength of your study, it might be more appropriate for the discussion section.

**Response:**

We have moved the NHI enrollment information to the discussion section. In addition, the enrollment figure has been updated to 99.9% according 2023 data.

- Similarly, lines 129-136 discuss the challenge of determining appropriate criteria for designing insurance products. This context could be better placed in the introduction, where you introduce the problem you aim to address.

**Response:**

We have moved the text on challenge of determining appropriate criteria for designing insurance products to the Introduction section.

- Be mindful of similar issues throughout the article and in the results section.The structure of the paper should follow the conventional order of Materials and Methods, Results, Discussion, and Conclusions. This would make the paper more coherent and easier to follow. Currently, the paper seems to mix the discussion with other sections, which reduces the impact of the main findings.

**Response:**

We have revised the manuscript to ensure that the discussion is not mixed with other sections.
